# Transformation of the long-term care system in Poland in the light of the maps of health needs: Jan Olminski

**DOI:** 10.1093/eurpub/ckac130.216

**Published:** 2022-10-25

**Authors:** J Olminski, M Koziol-Rostkowska, A Smiglewska, M Strozyk-Kaczynska

**Affiliations:** Department of Analyses and Strategies, Ministry of Health, Warsaw, Poland

## Abstract

As the inevitable ageing of the population progresses, the pressure on the public health system increases. The predicted rise in the share of people aged 65 and more from 19% in 2020 to 33% in 2050 jeopardises the capacity of the long term care in Poland. It demands immediate actions in public policies to strengthen the system and to provide the silver generation with the proper and adjusted services. That topic is one of the main issues for the map of health needs, developed by the Ministry of Health in Poland. The analyses investigate the current and future state of this part of the system, e.g. the number of visits, average length of stay, types of services, care-giver support ratio. Conclusions drawn from the data allowed to formulate a number of challenges, which include:

1. levelling up the access to the services,
2. increasing the number of outpatient services, provided at home or close to the place of residence,
3. implementation of the eHealth solutions,
4. support for the informal care-givers.

In line with these information, the actions and strategic frameworks have been established at the national level and included:

1. strategic approach for deinstitutionalisation, in order to bring the long term care closer to the patients’ environment, to provide them with an optimal level of comfort and to make them self-reliant for as long as possible,
2. enhancement of the inpatient care through transformation of less occupied wards into long term wards in district hospitals.

**Key messages:**

- The Maps of Health Needs help to create national public policies and strategic frameworks aimed at coping with the foreseen challenges of aging society pressuring the healthcare system.
- The main challenge for the long term care in the future is to channel the available resources in the appropriate way.

